# Twenty-first century droughts have not increasingly exacerbated fire season severity in the Brazilian Amazon

**DOI:** 10.1038/s41598-021-82158-8

**Published:** 2021-02-23

**Authors:** R. Libonati, J. M. C. Pereira, C. C. Da Camara, L. F. Peres, D. Oom, J. A. Rodrigues, F. L. M. Santos, R. M. Trigo, C. M. P. Gouveia, F. Machado-Silva, A. Enrich-Prast, J. M. N. Silva

**Affiliations:** 1 Departamento de Meteorologia, Instituto de Geociências, Universidade Federal do Rio de Janeiro, Rio de Janeiro, 21941-916 Brazil; 2 Centro de Estudos Florestais, Instituto Superior de Agronomia, Universidade de Lisboa, 1349-017 Lisboa, Portugal; 3 Instituto Dom Luiz, Universidade de Lisboa, 1749-016 Lisboa, Portugal; 4 Instituto Português do Mar e da Atmosfera, 1749-077 Lisboa, Portugal; 5 Department of Thematic Studies–Environmental Change, Linköping University, 58183 Linköping, Sweden; 6 Present Address: Programa de Geociências (Geoquímica Ambiental), Instituto de Química, Universidade Federal Fluminense, Niterói, 24020-141 Brazil

**Keywords:** Environmental sciences, Natural hazards

## Abstract

Biomass burning in the Brazilian Amazon is modulated by climate factors, such as droughts, and by human factors, such as deforestation, and land management activities. The increase in forest fires during drought years has led to the hypothesis that fire activity decoupled from deforestation during the twenty-first century. However, assessment of the hypothesis relied on an incorrect active fire dataset, which led to an underestimation of the decreasing trend in fire activity and to an inflated rank for year 2015 in terms of active fire counts. The recent correction of that database warrants a reassessment of the relationships between deforestation and fire. Contrasting with earlier findings, we show that the exacerbating effect of drought on fire season severity did not increase from 2003 to 2015 and that the record-breaking dry conditions of 2015 had the least impact on fire season of all twenty-first century severe droughts. Overall, our results for the same period used in the study that originated the fire-deforestation decoupling hypothesis (2003–2015) show that decoupling was clearly weaker than initially proposed. Extension of the study period up to 2019, and novel analysis of trends in fire types and fire intensity strengthened this conclusion. Therefore, the role of deforestation as a driver of fire activity in the region should not be underestimated and must be taken into account when implementing measures to protect the Amazon forest.

## Introduction

Fires in the Brazilian Amazon (BAMZ) are of three main kinds: deforestation, pasture or cropland maintenance, and forest fires^1,2^. In deforestation fires, the residual biomass from forest clearing activities is piled up and burned for several hours or days. Maintenance fires are performed in previously cleared areas, mainly in pastures, to get rid of weeds and prevent shrub encroachment^3,4^. Unlike the two previous types, forest fires are unintended, typically escape from pastures^5^ and burn uncontrolled through the understory of degraded forests where selective logging has opened up the canopy and facilitated drying of the litter layer. These three kinds of fires, all of them anthropogenic, essentially encompass all vegetation fire activity in the BAMZ, where natural fires are almost inexistent. Although lightning discharges are a non-negligible cause of mortality for large trees^6^, they very rarely originate fires and when they do, these are small and unimportant^7,8^. Since the BAMZ (Fig. S1) had very limited evolutionary exposure to fire as an ecological factor, it is poorly adapted and gets severely affected by even low intensity burning^1,2^. Amazon biomass burning disrupts native plant communities, reduces forest carbon stocks, and releases greenhouse gases and aerosols to the atmosphere, all with profound direct and indirect impacts on the regional and global climate^9^.

Climate variability and change also play an important role in the biomass burning over BAMZ, especially in drought years^10–13^. In 2005, the lack of precipitation induced by warming of the tropical North Atlantic Ocean, associated to the Atlantic Multidecadal Oscillation (AMO), affected mainly the western BAMZ^14–16^ and promoted an anomalously extensive and severe fire season in the region^11^ (Fig. S2). In 2007, drought mostly affected the Brazilian Cerrado and southeastern BAMZ, leading to a record of fire activity in the region and in the adjacent eastern edge of the BAMZ^13^ (Fig. S2). In 2010, the co-occurrence of a positive El Niño-Southern Oscillation (ENSO) and AMO phases induced record drought^17,18^ and fire activity over the eastern and southern BAMZ^19^ (Fig. S2). The latest drought is the El-Niño-related mega-event of 2015 (Fig. S3) that affected about 80% of the Amazon Basin^14,20–22^; its impacts on fire activity (Fig. S2) will be specifically addressed in this study.

Deforestation has long been recognized as a key driver of vegetation burning in the BAMZ^3,4,23,24^ and is enhanced during droughts and periods of weak forest governance^2,25^. While considering that fire in the region is primarily a land management tool, the possibility was raised that climate change and the expansion of degraded forests might withdraw fire from human control and convert it into a major ecological disturbance^1^. A recent study, hereafter A2018^26^, proposed the hypothesis that fire activity in the BAMZ has decoupled from deforestation, since the decrease in the annual rate of forest loss observed during the twenty-first century was not accompanied by a comparable reduction in fire activity and in pyrogenic emissions. According to this hypothesis, carbon emissions in the BAMZ are increasingly associated with forest understory fires occurring in anomalously dry years, in particular during the mega-drought of 2015. The decoupling hypothesis was quickly picked up by several authors^27–32^ and is cited in the IPCC Special Report on Climate Change and Land^33^. A2018 discussed various indicators of decoupling between deforestation and fire: (i) an increasing trend in the annual number of active fires detected per unit area deforested; (ii) a decreasing trend in the coefficient of determination (R^2^) of the regressions of active fire counts on deforestation rates, across phases of the Plan for Prevention and Control of Deforestation in the Amazon (PPCDAm); (iii) lack of a significant trend in CO emissions; (iv) increasing exacerbation of fire season severity in drought years, namely in the extent of area burned by forest understory fires.

However, assessment of the decoupling hypothesis by A2018 relied on an active fire database that contained incorrect data. This database was superseded by a corrected version released in July 2018^34^, currently available from the Brazilian Space Research Institute (INPE). Considering the relevance of the decoupling hypothesis for environmental and socio-economic policy making, we revisit the problem using the corrected active fire database, complemented by additional independent evidence from fire radiative power and pyrogenic emissions. The first goal of our study is to reconceptualise the drought-induced fire-deforestation decoupling hypothesis and the exceptionality of the 2015 fire season in the BAMZ. The second goal is to reassess the extent to which the spatial–temporal variability of fires over the region was affected during a period when climate and land use were steadily changing.

## Methods

### Study area and data

This study was carried out over the Brazilian Amazon biome (Fig. S1) during 2003–2019, containing the period used by A2018 (2003–2015) as providing evidence of a strong fire-deforestation decoupling. The region has an area of 4,196,943 km^2^ and covers the Brazilian states of Amazonas (AM), Roraima (RR), Amapá (AP), Pará (PA), Acre (AC), Rondônia (RO), Mato Grosso (MT), Maranhão (MA), and Tocantins (TO).

Due to problems that affected near real-time active fire detection, including MODIS image reception, ancillary data download, and metadata cataloging, INPE replaced its active fire database covering the period 1988 to November 2017 with a new database^34^. Henceforth, we will refer to the error-affected, replaced database, which was the one used by A2018, as the "superseded database", and to the new, corrected database, as the "current database". Both versions rely on information extracted at 1 km resolution from daily AQUA MODIS data (available at http://queimadas.dgi.inpe.br/queimadas/portal). Changes from the superseded version of the database included the adoption of collection 6 MODIS active fire detection algorithm and data, and the correction of the fire pixels detected by MODIS. Released by INPE in July 2018^34^, the current version of the database is now fully compatible and integrated with the NASA MODIS Collection 6 fire product^35^. The superseded database was used in A2018 but, as we demonstrate in this work, that version led to an underestimation of the decreasing trend in fire activity and to an inflated rank of year 2015 in terms of active fire counts over the period 2003–2015.

We further used fire radiative power (FRP)^36^ data, also extracted from Collection 6 fire product, with the same temporal and spatial resolution of active fires. We characterized deforestation patterns over the BAMZ with annual maps of progressive forest loss (hereafter deforestation) provided by the Program for Deforestation Assessment in the Brazilian Amazonia (PRODES)^37^ from 2003 to 2019, except where noted. We also used a map of tree cover in the year 2000 available from the Global Forest Change (GFC) product^38^. Both deforestation and tree cover datasets derive from Landsat 30 imagery, and were aggregated to match the active fire data 1 km-grid. Finally, we used annual means of monthly concentration of carbon monoxide at 800 hPa, hereafter [CO], from 2003 to 2015, that were obtained from the CO product version 8, derived from the MOPITT (Measurements of Pollution in the Troposphere) instrument^39^.

### Reassessment of the decoupling hypothesis

As in A2018, our analysis started by focusing on (i) annual time series of active fire counts; (ii) time series of the annual number of active fires detected per unit area deforested; (iii) regressions between the annual deforestation rate and the annual number of active fires, for the three phases of the PPCDAm^40^ (Phase I: 2004–2008, Phase II: 2009–2011, Phase III: 2012–2015) and for the full period, 2004–2015; (iv) maps of the extent of the active fire count anomalies for the 2003–2015 period. First, we relied on active fire counts from the superseded database used in A2018, and then we repeated the analysis with the current database, for the same study area and period (BAMZ, 2003–2015). Then, we performed a comparative analysis of results obtained with the two versions of the database and assessed the impact of the differences on the decoupling hypothesis. We use the full period (2003–2019) of the current database, except when revisiting the decoupling hypothesis. In this section, the procedures described in A2018 are replicated using both the superseded and the current databases restricted to 2003–2015; results from the analysis of the period 2003–2019 are presented as SM.

Our reassessment included two additional analyses. First, we assessed the [CO] trend and the extent to which interannual variability of [CO] is explained by annual deforestation rates. Then, we developed a time series of annual active fire counts discriminated by major fire type, aiming to clarify the relative frequencies of deforestation, maintenance, and forest fires, with emphasis on drought years 2005, 2007, 2010, and 2015. The approach proposed by Morton et al*.*^41^ was adopted to identify persistent fires by analyzing the number of cases with two or more active fire detections at the same pixel in a given year, from 2003 to 2019. Here, fire type classification relied on a set of thresholds imposed on fire persistence, forest cover, annual deforestation rates, and cumulative deforestation, applied to the active fire pixels in each year from 2003 to 2019. Persistent fires, i.e. active fires detected on two or more days on a given pixel and dry season, are often related to active deforestation, where fuels are piled up and burned repeatedly^4,42,43^. By contrast, non-persistent fires, i.e. active fires detected only once on a given pixel and dry season are typical of pasture management and burning of crop harvest residues, where the vegetation is fully consumed in a short period of time^41,44^. Accordingly, detections of two or more active fires in pixels with annual deforestation rate greater than or equal to 5% were classified as deforestation fires, this threshold corresponding to the minimum area of deforestation that the PRODES product is able to detect. Detections of three or fewer active fires on a pixel were classified as forest fires if tree cover in the year 2000 was greater than or equal to 70% and the cumulative deforestation from 2001 up to active fire detection year was lower than or equal to 20%. These cases represent fire detections in pixels with predominance of forest cover and limited land use change, and thus were classified as forest fires. The remaining cases were classified as pasture or cropland maintenance fires.

We then analyzed statistical distributions of FRP by fire type and by year, as a means of independent assessment of the fire type classification, and to characterize temporal patterns of fire intensity.

Finally, we applied principal component analysis (PCA) to each of the three datasets of annual maps of forest, maintenance and deforestation fires, in order to uncover patterns of variability in fire activity that provide insights into the relationships between fire, deforestation and droughts. Each pattern identified an independent mode of variability that explains a given fraction of the total variance of the original dataset. In order to be comparable, all datasets were previously standardized by subtracting the 2003–2019 mean from annual fire counts at each pixel and then dividing by the standard deviation for the same period.

## Results

### Revisiting the decoupling hypothesis

**Figure 1:**
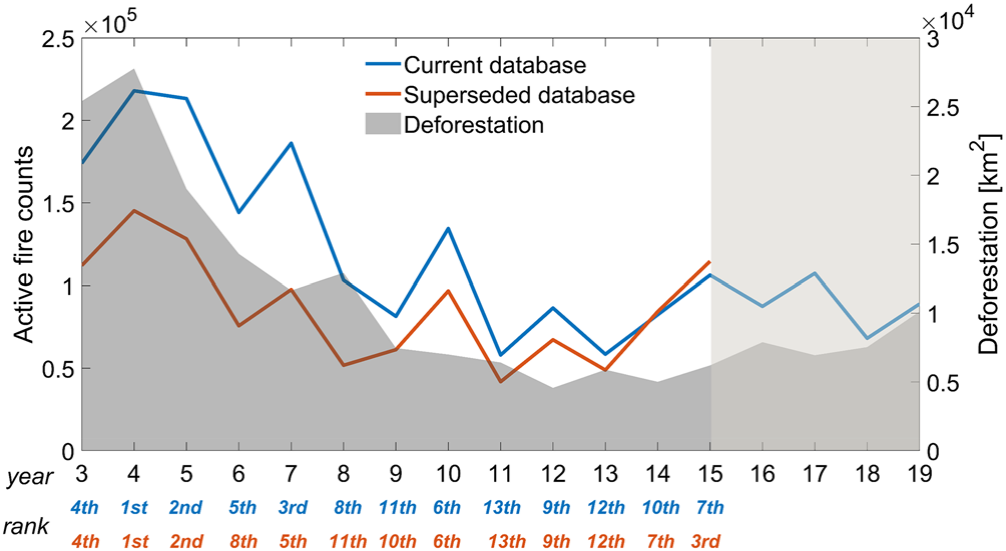
Interannual variability of fire activity and deforestation in the region during 2003–2019. Annual MODIS/AQUA active fire counts, left y-axis, for the current database (blue line) and for superseded database (red line) time series over the BAMZ. Each year is ranked by number of active fire counts (in blue for the current and in red for superseded databases) from 2003 to 2015. PRODES deforestation rates are depicted as the background gray area, right y-axis. Trend significance was analyzed with a two-tailed Mann–Kendall test of significance at the 0.01 level, and trend slopes were estimated using the non-parametric Theil-Sen robust linear regression.

Figure 1 compares the active fire time series from the superseded database and the current database, for the BAMZ. The time series of annual active fire counts in the superseded database displays a decreasing trend of − 3705 + /− 5702 counts year^−1^, which is more than three times weaker than the trend of − 11,625 + /− 6476 counts year^−1^ estimated from the current database in the 2003–2015 period. The superseded database data also ranks recent years higher in number of active fires than the current database, namely the year 2015, which ranks 3rd highest in the superseded database, but only 7th highest in the current database*.* Moreover, the number of fire counts for 2015 in the current database is 16% lower than the 2003–2015 average, and 50%, 42%, and 21% lower than those from the 2005, 2007, and 2010 drought years, respectively (Fig. 1). In turn, in the superseded database the values for 2015 were 36% higher than the 2003–2015 average, 10% lower than those for 2005, and 17%, and 18% higher than those for 2007, and 2010, respectively. The weakly negative trend in the superseded database time series appeared to strengthen the fire-deforestation decoupling hypothesis, because the decrease in fire activity did not follow the strong decline observed in annual deforestation rates.

**Figure 2:**
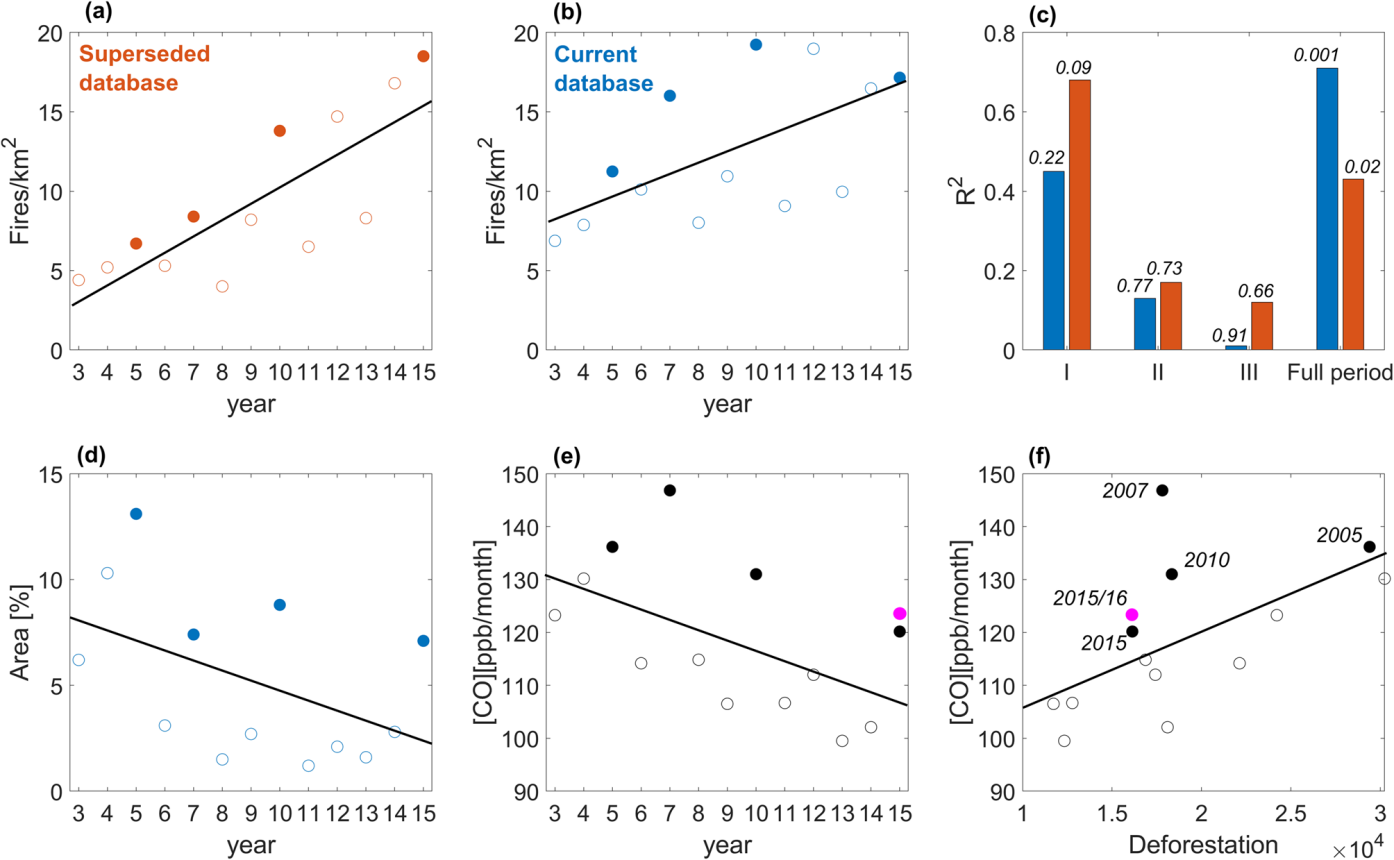
Time series of the annual number of active fires per km^2^ deforested from (**a**) the superseded database (R^2^ = 0.63; *p* value = 0.001) and (**b**) the current database (R^2^ = 0.35; *p* value = 0.03). (**c**) R^2^ for fire-deforestation regressions and respective *p* values (on top of bars) for the full period (2003–2015) and as in A2018 for the three PPCDAm (Phase I: 2004–2008, Phase II: 2009–2011, Phase III: 2012–2015). (**d**) Percent area of the BAMZ with active fire count anomalies in excess of 2 standard deviations using the current database. (**e**) Time series of the annual mean of monthly [CO] at 800 hPa (R^2^ = 0.32; *p* value = 0.043). (**f**) Regression of mean monthly [CO] at 800 hPa on annual deforestation rates from the Global Forest Change (GFC) product^38^ (R^2^ = 0.27; *p* value = 0.07). Full dots in panels (**a**), (**b**), (**d**), (**e**), and (**f**) indicate drought years (2005, 2007, 2010 and 2015). The magenta dot in panels (**e**) and (**f**) represents active fire counts (the current database) for April 2015 to March 2016.

Using data from PRODES^40^, time series of number of active fires per km^2^ deforested were then derived using active fire counts from the superseded database (Fig. 2a) and the current database (Fig. 2b). High values of this ratio indicate an excess of fires beyond those associated with deforestation, suggesting decoupling. The number of active fires per km^2^ deforested displays a positive and significant trend in both cases, but in the current database (Fig. 2b) time explains only half as much of the variance in the number of fires per km^2^ than in the superseded database (Fig. 2a). In Fig. 2b, 2015 presents the third highest, rather than the highest value of the ratio. There is still an increasing trend in the fire number per deforested area, suggestive of some degree of decoupling, but the increase is not nearly as strong as in the superseded database and is barely significant at the 95% confidence level. Except for 2012, Fig. 2b also shows a clear separation between drought and non-drought years. The high value of the ratio in 2012 results from the combination of the lowest deforestation rate of the entire study period (Fig. 1) with localized droughts conditions^30,45^ that contributed to a local spike in the time series of fire activity.

Figure 2c compares the coefficient of determination (R^2^) and significance values obtained for the regression of active fire counts derived from both databases on PRODES deforestation rate, for the BAMZ. Considering the very small number of years in the regressions for each phase of the PPCDAm, namely Phase I: 2004–2008; Phase II: 2009–2011 and Phase III: 2012–2015, we tested them for significance and found all to be non-significant, except for the full PPCDAm period (2003–2015). Therefore, results do not indicate a significant decrease in the explanatory power of annual deforested area for the number of active fires over the three PPCDAm phases. Results for the period 2003–2015 show that the annual deforestation rate explained 68% of the variance in active fire counts in the BAMZ, substantially higher than the 43% obtained with the superseded data, indicating stronger coupling between deforestation and fire than suggested by A2018.

Results obtained with the superseded database also had a strong impact on the assessment of the spatial extent of active fire count anomalies in the BAMZ during the drought years. A time series for that variable derived from the current database (Fig. 2d) and the respective maps (Fig. S2) show that the spatial extent of the 2015 anomaly is similar to those of 2010 and 2007 and 54% of that for 2005, and not twice as large, as A2018 had estimated. Again, when using the superseded database, the extreme character of the 2015 fire season was exaggerated. With the current database there is a non-significant trend in the spatial extent of the active fire anomalies larger than two standard deviations in the BAMZ. After 2005, the data clearly separate between drought and non-drought years (Figs. 2d and S4 c), and each group has almost stable fire anomaly areas, of around 9% and 2% of the biome, respectively. These results provide evidence of droughts exacerbating fire activity over the BAMZ, but also clearly indicate that the magnitude of this effect has not increased during the twenty-first century.

A2018 relied on pyrogenic emissions data as additional evidence of decoupling between fire activity and deforestation. They found that the total atmospheric column [CO] measured by the MOPITT instrument displayed no significant trend over the period 2003–2015. Since deforestation dropped significantly over the same period, the lack of a decreasing trend in [CO] was considered evidence of decoupling. However, when we use annual means of monthly [CO] at 800 hPa over the same period, there is a marginally significant negative trend, where time explains 32% of the variance in [CO] (Fig. 2e). In turn, annual deforestation rates display a non-significant relation with [CO] at 800 hPa (R^2^ = 0.27, *p* value = 0.07) (Fig. 2f). When the late onset of the 2015 fire season^14,46^ was taken into account by extending the [CO] data until March 2016, the resulting slight increase in the corresponding value renders the [CO] trend insignificant at the 95% confidence level. When the same adjustment was applied to the regression of [CO] on the annual deforestation rate it remained non-significant. However, savanna and agricultural fires in the Arc of Deforestation and in the adjacent Cerrado, namely in regions that export emissions to Amazonia, made unusually large contributions to biomass burning during the 2007 and 2010 fire seasons^47^. Both years registered the two highest numbers of active fires^48^, fire emissions^49^ and burned area^50^ in Cerrado, which were mainly associated with extreme droughts in the region^51^. We assessed the effect of the anomalously high transport of pyrogenic emissions into the Amazon in 2007 and 2010 on the [CO] trend with a sensitivity analysis. Such emissions may account for up to half of the atmospheric optical depth during the dry season^52,53^, a pattern that also applies to CO^54^. The sensitivity analysis was performed by decreasing the [CO] values in 5% increments whereby we found that a 10% reduction in [CO] for the years 2007 and 2010 was sufficient to yield a significantly decreasing [CO] trend at the 95% confidence level (*p* value = 0.02), where time explains 40% of the variance in [CO]. The regression of [CO] on annual deforestation rate (Fig. 2f) also became significant at 95% (R^2^ = 0.44, *p* value = 0.01).

Extension of this analysis up to 2019 reinforces the results obtained above. The variance in the total number of active fires explained by deforestation remained constant at 68% (Fig. S4a); the marginally significant positive (negative) trend in the number of active fires per km^2^ deforested (spatial extent of active fire count anomalies) is no longer significant at the 5% level (Fig. S4b).

### Patterns of active fire variability

**Figure 3:**
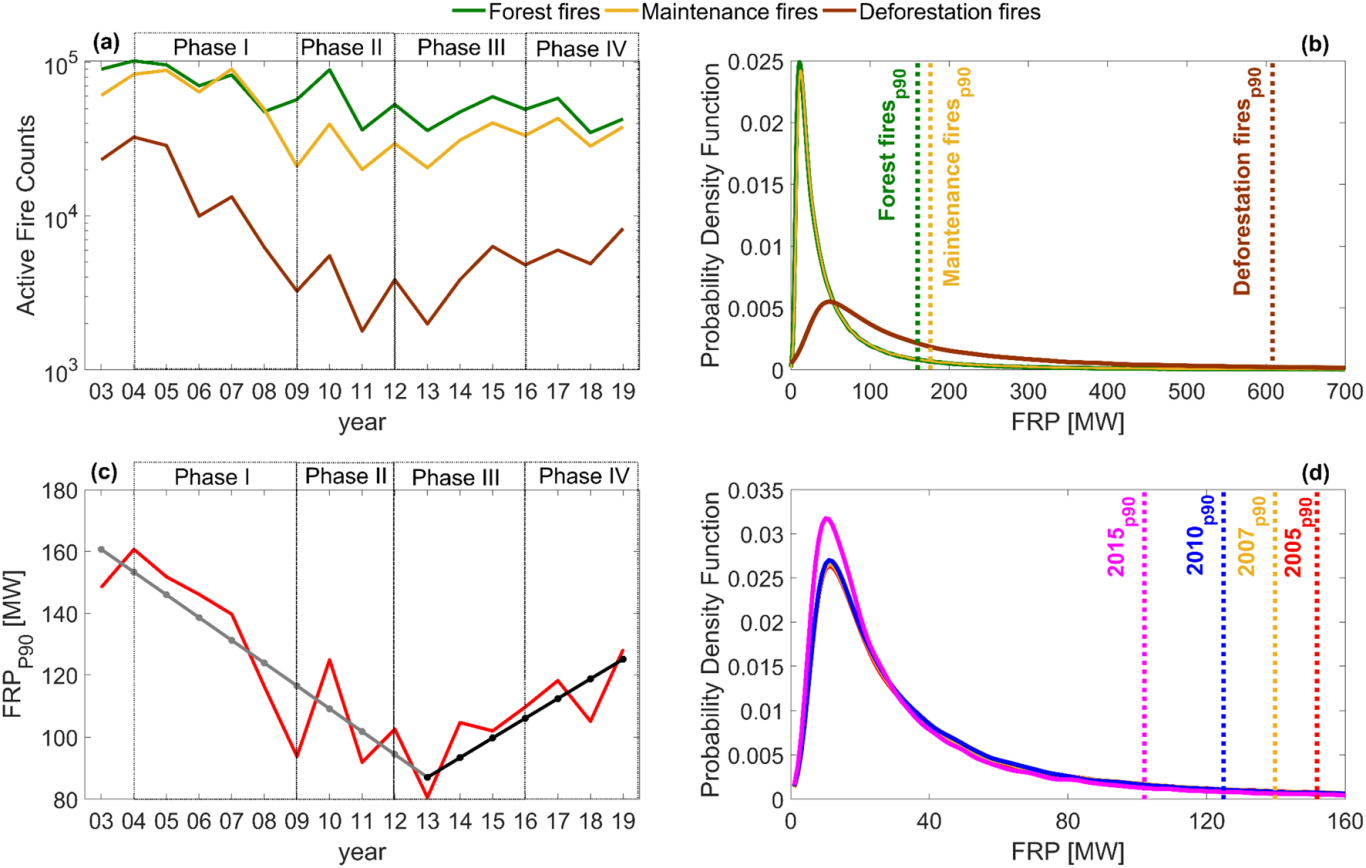
(**a**) Interannual variability of active fires classified as forest fires (green), maintenance fires (orange), and deforestation fires (brown) for the BAMZ; (**b**) Probability density function of FRP for each fire type from 2003 to 2019; (**c**) Interannual variability of the 90th percentile of FRP and the linear regression model with a break point representing the best minimum square fit of all models (with break points between 2005 and 2017). Trend significance was analyzed with a two-tailed Mann–Kendall test of significance at the 0.05 level, and both the decreasing (2003–2013) and the increasing (2013–2019) branches are significant; (**d**) Probability density function of fire radiative power (FRP) for drought years 2005, 2007, 2010, and 2015. Vertical lines indicate the 90th percentile of FRP for each (**b**) fire type; (**d**) year.

**Figure 4:**
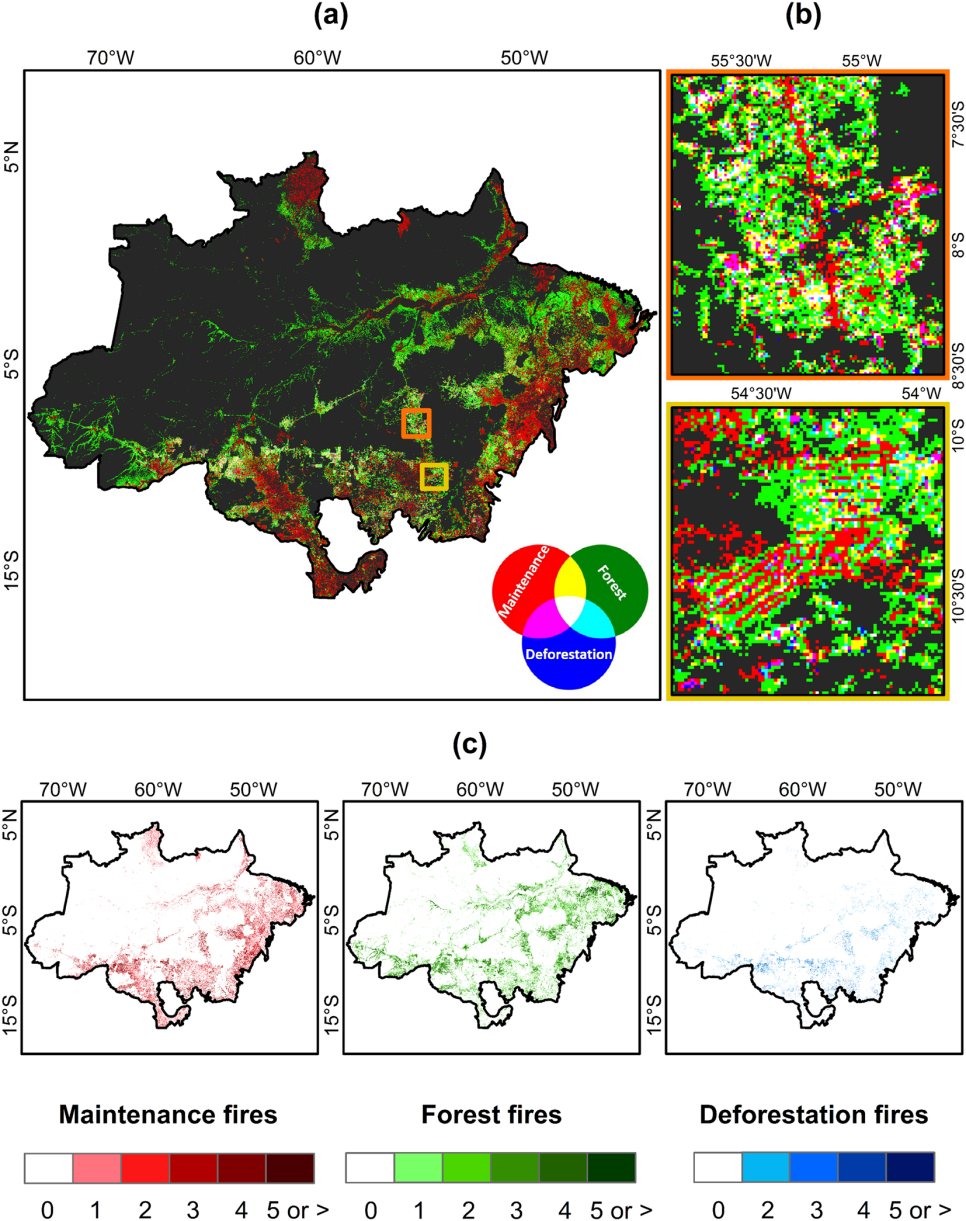
(**a**) RGB composite of fire types for the period 2003–2019 at 1 km resolution; (**b**) insets showing spatial detail of fishbone deforestation and subsequent use for an area in Northern Mato Grosso (yellow rectangle) and Southwestern Pará (orange rectangle); (**c**) cumulative fire counts of maintenance, forest, and deforestation fires over the period 2003–2019. Colour scales show the number of active fire detections in each 1 km pixel. In panels (**a**) and (**b**) we have used Python and QGis open-source platforms.

Although a biome-scale analysis as performed in the previous section was required to clarify the impact on the decoupling hypothesis of the use of flawed data by A2018, the large geographical extent and spatial heterogeneity of the BAMZ has to be taken into account to avoid overgeneralized conclusions about trends and drivers^55^. Figure 3a displays the annual time series of fire events (2003–2019), stratified into deforestation, maintenance and forest fires according to the number of active fires detected in a given pixel and year, and the tree cover and cumulative deforestation in that same pixel (see Methods). Deforestation, maintenance, and forest fires respectively represent 8%, 39%, and 53% of the total number of active fires in 2003–2019, a result that is very close to the values of 9%, 41%, and 50% obtained in a previous study^56^ for the period 2003–2010 (Fig. S5) that used a supervised classification approach. The effects of droughts (2005, 2007, 2010, and 2015) on fire activity are evident, especially on forest fires and maintenance fires. These two fire types displayed very similar numbers in the 2005 and 2007 droughts, but the 2010 drought preferentially exacerbated the occurrence of forest fires, which accounted for 65% of that year’s total. Our fire type classifications (Fig. 3a) are also consistent with previous studies^56^ and show that forest and maintenance fires are the most abundant types (Fig. 4). Since fires in deforested areas soon become maintenance fires, only very recent deforestation fires where no forest fires had previously occurred will show up in the maps (Fig. 4b). Deforestation fires are fewer and more concentrated along the Arc of Deforestation, while maintenance and forest fires display very similar patterns because most forest fires are located less than 1 km from the forest edge^5^ (Fig. 4c).

Fire intensity, represented by FRP, varies with fuel type, size, and moisture content^57,58^. Figure 3b displays FRP probability density functions and identifies the 90th percentile of FRP (henceforth, FRP90) for the three fire types. Most fires display low intensity and, therefore, mean or median FRP values provide very limited discrimination between fire types. However, the FRP distributions for some fire types (e.g. crop harvest residue burning) have light tails, while those of consuming large amounts of woody fuels (e.g. forest slash burning) display much heavier tails. Therefore, high percentiles of the FRP probability density functions improve the discrimination of fire types^59^. Forest and maintenance fires display very similar FRP distributions and FRP90 values, while deforestation fires are much more intense, with a heavier right tail and FRP90 almost four times higher than that of forest fires (Fig. 3b). Since we did not use FRP to classify fire types, these results supply convergent evidence for the results of our classification.

In agreement with the decrease in annual deforestation rates observed by a previous study^60^, the timeseries of FRP90 (Fig. 3c) shows a marked decrease from 2003 to 2013 (after the end of Phase II of the PPCDAm), followed by an increase that extends to 2019. Both the decreasing and the increasing segments of the trend are significant at the 0.05 level. FRP90 for the four drought years decreased monotonically and the value in 2015 is about 2/3 of that observed in 2005 (Fig. 3d). Again, drought-induced fire exacerbation in 2015 was the mildest of the four droughts of the twenty-first century (Fig. 3d).

We further analysed the hypothesis that drought conditions are not increasingly exacerbating fire activity in the BAMZ and that fire activity during the extreme drought of 2015 was not exceptional by studying the variability of fire activity using PCA applied to standardized datasets of forest, maintenance, and deforestation fires. This technique allows decomposing the variability of the three types of fire activity into independent modes, highlighting spatial and temporal differences that cannot be uncovered when the analysis is performed at the biome level.

**Figure 5:**
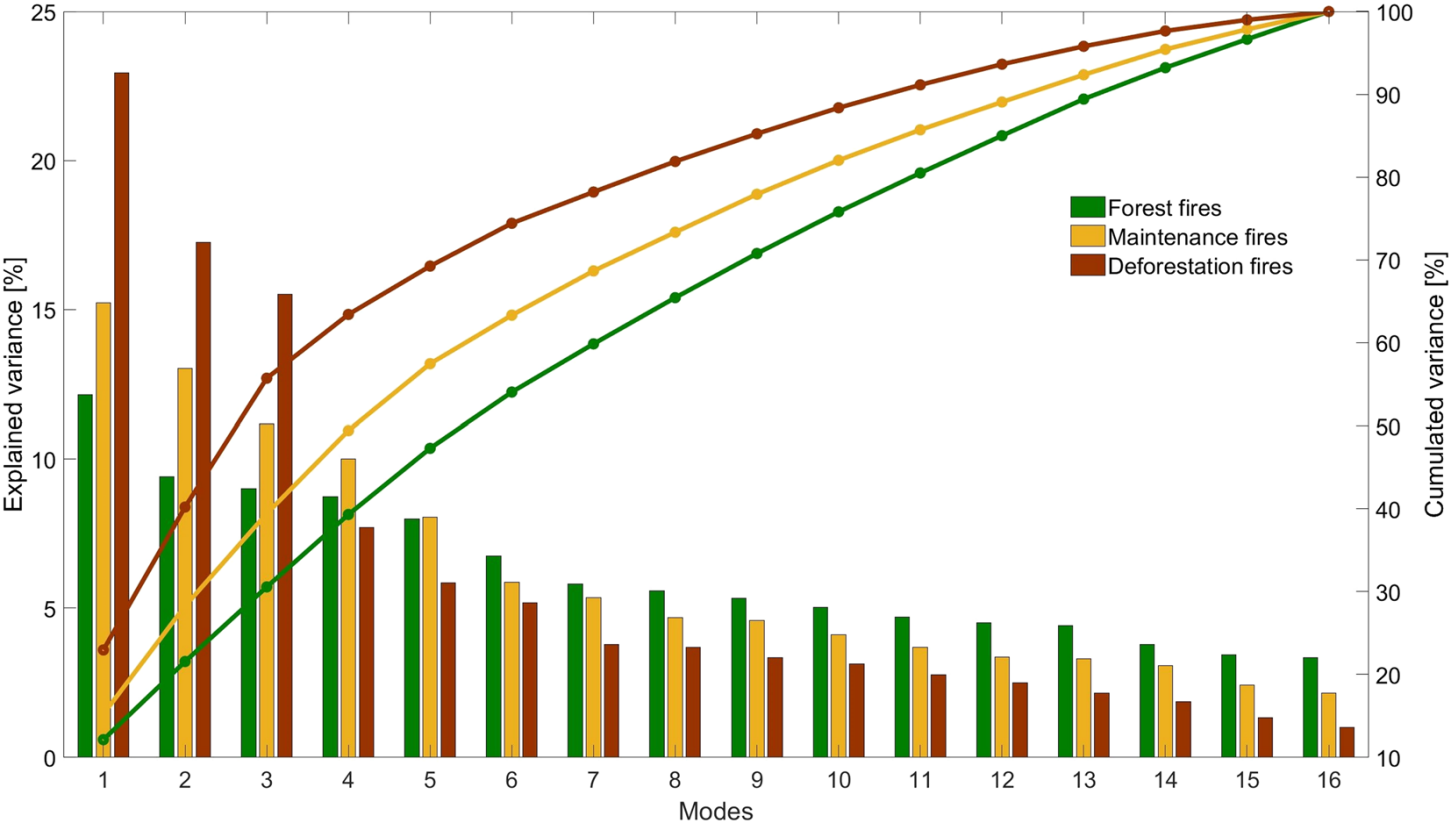
The variances explained by the different modes for each type of fire activity: forest (green), maintenance (orange) and deforestation fires (brown) for the 2003–2019 period.

**Figure 6:**
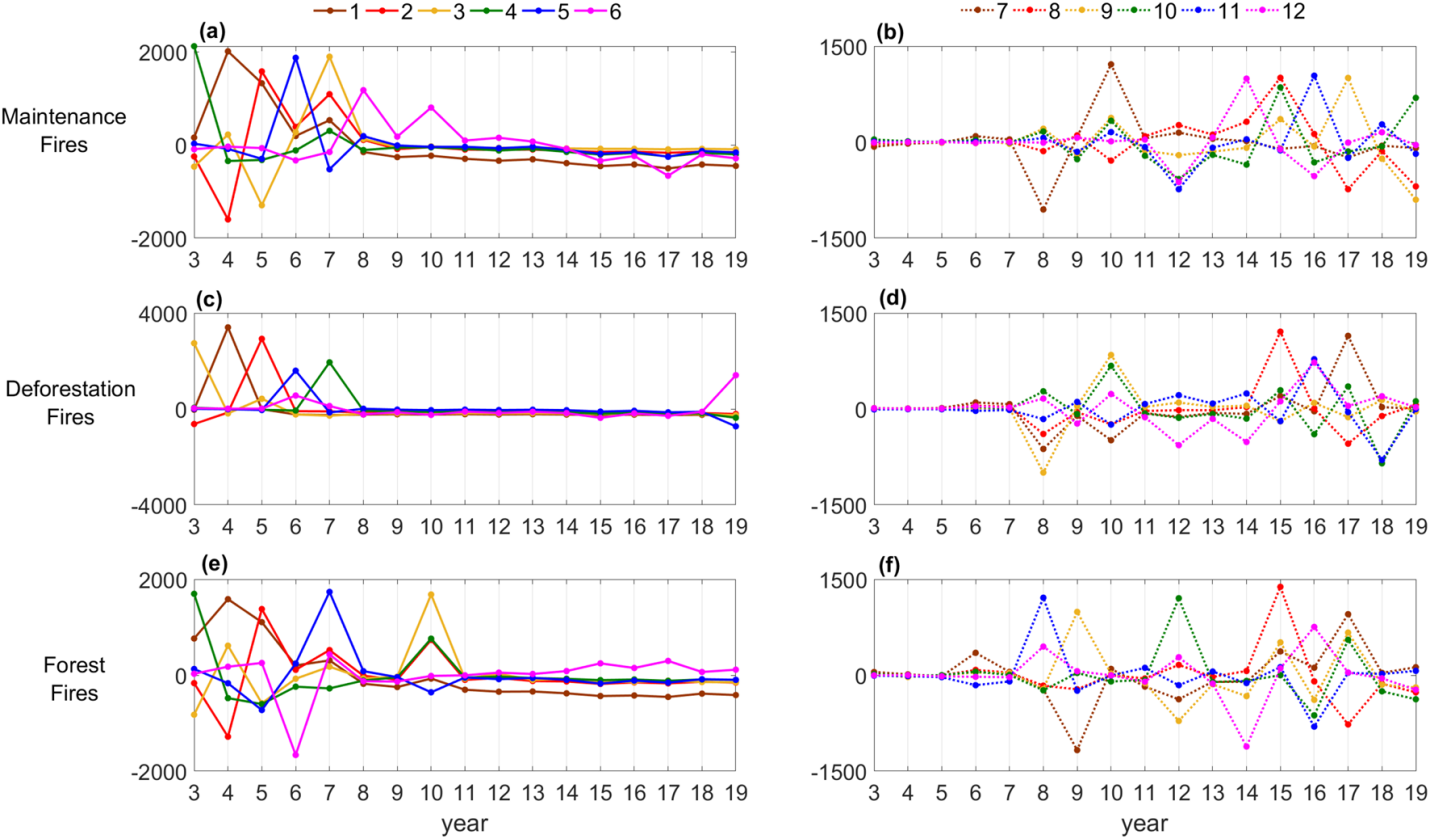
Time series of PCs 1–6 for (**a**) maintenance, (**c**) deforestation, and (**e**) forest fires, and of PCs 7–12 for (**b**) maintenance, (**d**) deforestation, and (**f**) forest fires for the 2003–2019 period.

Reflecting the stronger spatial consistence of deforestation fires through time, when compared to maintenance fires, and especially to forest fires, the first six modes respectively explain 74%, 63% and 54% of the total variability in deforestation, maintenance, and forest fires (Fig. 5) and, when considering the first twelve modes, the amounts of explained variance raise up to 93%, 89% and 85%. Time series of PCs 1–12 for the three types of fire activity (Fig. 6) reveal two contrasting subperiods, from 2003 to 2007 and from 2008 to 2019. The first subperiod is characterized by large oscillations in PCs 1–6 that contrast with very weak changes in PCs 6–12, the second subperiod presenting opposite characteristics, with large oscillations occurring in PCs 7–12 and PCs 1–6 exhibiting virtually no oscillations except for the peaks of PCs 3 and 4 in 2010 for forest fires and the peak of PC6 in 2008 and 2010 for maintenance fires.

Time series of PC1 for the three types of fire activity reflect the long-term decline in fire counts (trend) and present similar characteristics, all with a well-defined maximum in 2004, followed by a steep decrease in the following years, especially abrupt in the case of deforestation fires (Fig. 6). The temporal variability is transferred from PCs 1–6 before 2008 to PCs 7–12 after 2008 reflecting a change in the variability of fires between the two periods. For all three types of fire activity the drought years are conspicuous in PCs 1–12, the events of 2005, 2007 and 2010 displaying peaks in one or more of PCs 1–6, in sharp contrast with the drought of 2015 that only presents a peak in PC8. Changes in the spatial patterns of variability of fire activity are in turn revealed by Empirical Orthogonal Functions (EOFs) of all fire types, especially in the first three modes that explain 56%, 39% and 31% of the variability of deforestation, maintenance and forest fires. As shown in Fig. S6, maps of the sign of EOFs 1–3 enhance the strong contrasts of fire activity in all three types between Southern and Northern BAMZ, as well as between the cores of areas of fire activity and the edges, where the most recent deforestation took place.

## Discussion

Results obtained using the superseded database led to an overestimation of the extent to which deforestation is losing explanatory power over the variance in fire activity in the BAMZ and to a spurious increasing trend in drought-induced exacerbation of fire over the period 2003–2015. When the analysis is replicated using corrected information from the current database, the evidence of fire-deforestation decoupling becomes weaker (trend in the number of fires per km^2^; trend in CO emissions) or disappears (variance explained by fire-deforestation regressions through the PPCDAm phases). In addition, we failed to detect increasing trends in fire season severity in drought years (trends in the number of active fires per km^2^ deforested and in the spatial extent of large positive fire count anomalies). Nevertheless, during drought years the number of active fires per km^2^ deforested is substantially higher than during non-drought years (all full circles are above the open circles in Fig. 2b). This indicates that although fires during drought years affect significantly more the forest than during non-drought years, such exacerbation effect is not increasing.

Our analysis of [CO] at 800 hPa revealed a marginally significant trend, which lost significance when the data were adjusted for the late onset of the 2015 dry season. However, introduction of a conservative 10% reduction to compensate for the anomalously high transport of pyrogenic emissions into the Brazilian Amazon in 2007 and 2010 yielded a significantly decreasing [CO] trend, and a significant regression of [CO] on deforestation rates, further weakening claims of fire-deforestation decoupling.

We also showed that the trend in the number of active fires per km^2^ deforested is marginally significant at the 95% confidence level. Therefore, it provides weak evidence in support of the fire-deforestation decoupling hypothesis. In defence of their associated hypothesis that forest understory burning in drought years was the main responsible for fire-deforestation decoupling, A2018 referred lack of evidence of an increase in pasture area burned relatively to forest area burned, which might explain decoupling. However, data from the Digital Atlas of Brazilian Pasturelands^61^, available from the Laboratory for Image Processing and Geoprocessing^62^ show that the ratio between annual deforestation rate and cumulative pasture area in the BAMZ dropped from 7% in 2003 to 1.6% in 2015. That in itself would certainly contribute to the decreasing proportion of deforestation as a fire cause in the BAMZ, and is corroborated by a previous study where a classification of fire types^56^ shows that between 2004 and 2010 the ratio of deforestation fires to forest fires dropped from 30 to 4%. Over the same period, the ratio of deforestation fires to maintenance fires decreased from 35 to 7%. Results from our own active fire type classification (Fig. 3a) reveal decreases in the ratio of deforestation fires to forest fires (deforestation fires to maintenance fires) from a maximum of 32% (39%) in 2004 to a minimum of 5% (9%) in 2011, followed by an increase up to 19% (22%) in 2019. The declining ratios of deforestation fires to maintenance fires reveal an important contribution of maintenance (mostly pasture) fires to fire-deforestation decoupling in the Brazilian Amazon. However, the relative share of deforestation fires out of the total number of fires increased again between the 2011 minimum of 3% and 2019, when it reached 9%, exceeding those of the second half of PPCDAm Phase I (6–7%).

The differences in FRP between fire types reflect the kinds and amounts of fuel burned. The high FRP90 value for deforestation fires corresponds to the combustion of heavy loadings of woody forest harvest residues. The much lower values for maintenance fires result from the combustion of lighter loadings of fine, predominantly herbaceous fuels^63–65^. Forest understory fires primarily consume litter fuel, with loadings similar to those of pastures, and spread very slowly, with low intensity^44^.

Results from PCA performed on forest, maintenance and deforestation fires contribute to consolidate the hypothesis that drought conditions are not increasingly exacerbating fire activity in the BAMZ and that fire activity was not exceptional during the extreme drought of 2015. By decomposing the variability of the three types of fire activity into independent modes of variability, PCA highlights spatial differences that were obscured in the analyses performed at biome level and allows comparing the spatial and temporal consistency of the various types of fire activity. Time series of PCs revealed differences in temporal variability of the three types of fires between the period 2003–2008, when the variability is captured by PCs 1–6, and the period 2009–2019, when it is captured by PCs 7–12. This is especially interesting because 2008 is the end of the Phase I of PPCDAm, which marks the transition from extensive deforestation, predominantly in large patches, to lower levels of deforestation, mostly in small patches^66,67^. Time series of PCs 1–12 also evidenced the decreasing trend in fire season exacerbation during the drought episodes of 2005, 2007, 2010, and 2015. Changes in spatial variability of fire activity were in turn highlighted by the signs of EOFs 1–3 that show contrasts between Southern and Northern BAMZ, and between the cores and borders of the areas of fire activity.

The strong link of deforestation fires and maintenance fires with human activities reflects on the number of modes concentrating most of the variability, with about two thirds of the total variance explained by the first four modes for deforestation, six modes for maintenance, and eight modes for forest fires. This feature translates the concentration in space and the regular pace in time of the use of fire in human activities, especially for deforestation fires, contrasting with forest fires, which are susceptible to sporadic weather extremes. The time series of PCs for the three types of fire activity present a shift in variability from modes 1–6 to modes 7–12. The four droughts left different fingerprints in the main modes of fire variability. While the drought of 2005 is conspicuous in PC2 for all three types of fire activity, the following events are mostly reflected in progressively higher modes, which explain less variability. The 2007 drought translates into peaks of PC3, PC4 and PC5 for maintenance, deforestation and forest fires, respectively. The 2010 drought only emerges on PC3 and PC4 for forest fires, on PC6 for maintenance fires and is barely noticeable for deforestation fires (that are related to human practices modulated by law enforcement). Contrasting with the previous drought years of 2005, 2007 and 2010, the year of 2015 just reflects on mode 8 for all types of fire activity, a strong indication that the impact of drought conditions was moderate, especially in the case of deforestation and maintenance fires, which although dependent on meteorological conditions, mostly result from human activities.

A recent study^54^ also found evidence of decoupling in the fact that the sharp decline in deforestation trends in Brazilian Amazonia over the period 2001−2016 was not matched by trends in fire-related variables (active fire counts, burned area, aerosol optical depth and CO_2_ emissions), which were much less significant. The authors reported a non-significant trend for active fire counts, which contrasts with our finding of a significantly decreasing trend. However, they only used active fires with confidence level above 80%, a drastic filtering that precludes a direct comparison with our results. These authors obtained a non-significant trend in area burned, and a non-significant relationship between burned area and deforestation, but the adequacy of using Global Fire Emissions Database, version 4 burned area data to analyze Amazon tropical deforestation is questionable. For the MODIS era, those data are based exclusively on the MCD64A1 500 m burned area product^68^, which is notoriously inaccurate over tropical forests, with omission error of 91% and commission error of 64%^69^. Therefore, these burned area data are unlikely to provide an accurate characterization of vegetation burning and, therefore, of fire-deforestation decoupling in the Amazon. Nevertheless, their aerosol optical depth (AOD) data and CO_2_ emissions clearly identify two distinct periods in the relationship between deforestation and fire, suggesting decoupling^54^. However, as far as we can tell, the analysis did not take into account the anomalously high import of emissions from the Cerrado in the 2007 and 2010 years^47^. Therefore, the claim that AOD and CO_2_ data provide evidence of decoupling may be weaker than suggested.

The relationships between fire, land use, and climate in the BAMZ are very dynamic, and during the period of our analysis there were substantial declines in annual deforestation rates and in fire activity, a substantial expansion in the area of pasturelands, and four droughts that exacerbated fire to various extents. The role played by deforestation resulting from the strong anthropogenic pressure the region experienced during the last decades, has led to land use change associated with forest conversion into agriculture and pasture, selective logging, and forest fragmentation^70–72^. From 2000 to 2013 in the Brazilian Amazon, 72% of total deforestation was related to agro-industrial land clearing for pasture (63%) and cropland (9%)^13^. The observed decrease in deforestation in the BAMZ since 2004 can be primarily attributed to effective environmental legislation together with declining prices of export commodities^4,72,73^.

The PPCDAm was introduced in 2004 and during its Phase I, which lasted until 2008, inter-ministerial policies went into effect aimed at curbing annual deforestation rates down from a mean annual baseline over the previous decade of over 19,342 km^2^. This phase of the PPCDAm was considered the most successful at significantly decreasing rates of illegal deforestation by employing command-and-control strategies, informed by a newly satellite-based system for near real-time deforestation monitoring introduced by INPE. Although the mean annual rate of deforestation over the five years of Phase I remained very high, at 17,127 km^2^, it decreased from 27,772 km^2^ in 2004 to 12,911 km^2^ in 2008. Fire activity remained high during this period, which recorded two droughts in five years, with a mean annual value of 172,916 active fires. During the three years of the PPCDAm Phase II (2009−2011), there was one drought in 2010, mean annual deforestation rate decreased further to 6961 km^2^ and the mean annual number of active fires dropped to 91,288. During Phase III of the PPCDAm (2012–2015), there was a single drought, in 2015, deforestation dropped to 5420 km^2^ per year and the mean annual number of active fires further decreased to 83,409. Finally, during the Phase IV (2016–2019), deforestation increased to 8126 km^2^ per year and the mean annual number of active fires further increased to 87,969. The decrease in fire activity from Phase I to Phase III of the PPCDAm appears to reflect not only the falling deforestation rates, but also the decreasing proportion of drought years in each phase. In terms of individual droughts, low deforestation rates seem to dampen the drought-induced exacerbation of fire activity. The increase in deforestation rates during PPCDAm Phase IV enhanced again the sensitivity of fire activity to drought, such that the total number of fires during the moderate 2017 drought^30^ was equivalent to that observed during the much more severe 2015 event.

## Conclusions

Our analysis of the relationships between deforestation, fire, and droughts in the BAMZ revealed evidence of some fire-deforestation decoupling, but not as strong as previously suggested by A2018, who relied on information from a flawed dataset. Weakened evidence of decoupling is conveyed by trends in the number of active fires per km^2^ deforested and in the concentration of pyrogenic emissions. Classification of active fires into the three major types that occur in the BAMZ showed that maintenance fires play an important role in fire-deforestation decoupling, as would be expected from the substantial expansion of pasture area, mainly between 2003 and 2008. Their occurrence was exacerbated by drought to a similar extent with that of forest fires during the 2005 and 2007 events, and slightly below during the 2015 mega-drought. In 2010, the drought-induced positive fire anomaly was strongly dominated by forest understory fires.

We did not find evidence that twenty-first century droughts are increasingly exacerbating fire seasons in the BAMZ. On the contrary, the mega-drought of 2015 had the mildest impact of all four droughts on the corresponding fire season, even when adjusted for its late onset and continuation up to March 2016. This was attested by the time series of active fire data, of the annual ratio of active fires to area deforested, and of the percent area of the BAMZ displaying active fire count anomalies larger than two standard deviations. Results from principal component analyses of annual maps of deforestation, maintenance, and forest fires at 1 km spatial resolution thoroughly confirmed the biome-level conclusions.

We believe that our results will be of use to researchers and managers concerned with the Amazon region, at a time when both climate and land use are steadily changing^13,74^. To make matters worse, anomalies induced by ENSO and AMO are expected to continue impacting the Amazon, including through an increased frequency of extreme droughts^75,76^. Such an increase in hydroclimatic extremes, compounded with anthropogenic land cover changes are expected to further promote fire activity in this region^77,78^. Deforestation is now increasing again in the BAMZ, particularly in the central and northern half of the region where previously intact forests are being increasingly disturbed^79^. According to the PRODES satellite monitoring program, deforestation in the Brazilian Amazon attained a minimum of 4571 km^2^ in 2012 and since then has been oscillating on an upward trend, reaching 9762 km^2^ in 2019, the highest value since 2009 (http://www.obt.inpe.br/prodes/dashboard/prodes-rates.html#). This increase is also evident in the total number of fires as well as in FRP90, which has reached the level of 2010.

These recent increments in annual deforestation rate are being observed under the current Brazilian government, which is rolling back environmental legislation that protected native vegetation, open up the Amazon rainforest to agrobusiness, and weaken licensing requirements for construction of dams, roads and for mining permits^80–83^. The Supreme Court recently upheld the 2012 overhaul of the Brazilian law protecting native vegetation, which implied dropping penalties for illegal deforestation activities in the Amazon prior to July 2008^84–86^. The current USA–China trade war is also pointed as a driver of further deforestation in the Amazon due to the need to ramp up soybean production^87^. The ongoing haphazard economic and political scenario, combined with frequent and intense droughts, may expose the Amazon Basin region to unprecedented increases in the area affected by fire, leading to severe impacts at all levels. Therefore, deforestation control policies are crucial and should not be weakened.

## Supplementary Information

Supplementary Figures.

## Data Availability

All data are publicly available via the links indicated in the text.
