# Supplementary material for: Twenty-first century droughts have not increasingly exacerbated fire season severity in the Brazilian Amazon
Source: Sci Rep. 2021 Feb 23;11:4400. doi: 10.1038/s41598-021-82158-8 (PMC7902828; doi:10.1038/s41598-021-82158-8)
Supplement: Supplementary file 1 — Supplementary Figures. [file 41598_2021_82158_MOESM1_ESM.docx]

Supplementary Materials for:

21st century droughts have not increasingly exacerbated fire season severity in the Brazilian Amazon

R. Libonati^1,2,3*^, J. M. C. Pereira^2⸸^, C. C. DaCamara^3⸸^, L. F. Peres^1,4^, D. Oom^2^, J. A. Rodrigues^1^, F. L. M. Santos^1^, R. M. Trigo^1,3^, C. M. P. Gouveia^3,4^, F. Machado-Silva^1§^, A. Enrich-Prast^1,5^, J. M. N. Silva^2^

^1^Departamento de Meteorologia, Instituto de Geociências, Universidade Federal do Rio de Janeiro, Rio de Janeiro, 21941-916, Brazil.

^§^now working in the Instituto de Química, Universidade Federal Fluminense, Niterói, 24020-141, Brazil

^2^Centro de Estudos Florestais, Universidade de Lisboa, Lisboa, 1349-017, Portugal.

^3^Instituto Dom Luiz, Universidade de Lisboa, Lisboa, 1749-016, Portugal.

^4^Instituto Português do Mar e da Atmosfera, Lisboa, 1749-077, Portugal.

^5^Department of Thematic Studies–Environmental Change, Linköping University, Linköping, 58183, Sweden.

^*^ corresponding author [renata.libonati@igeo.ufrj.br](mailto:renata.libonati@igeo.ufrj.br)

^⸸^these authors contributed equally to this work

Figures S1–S6


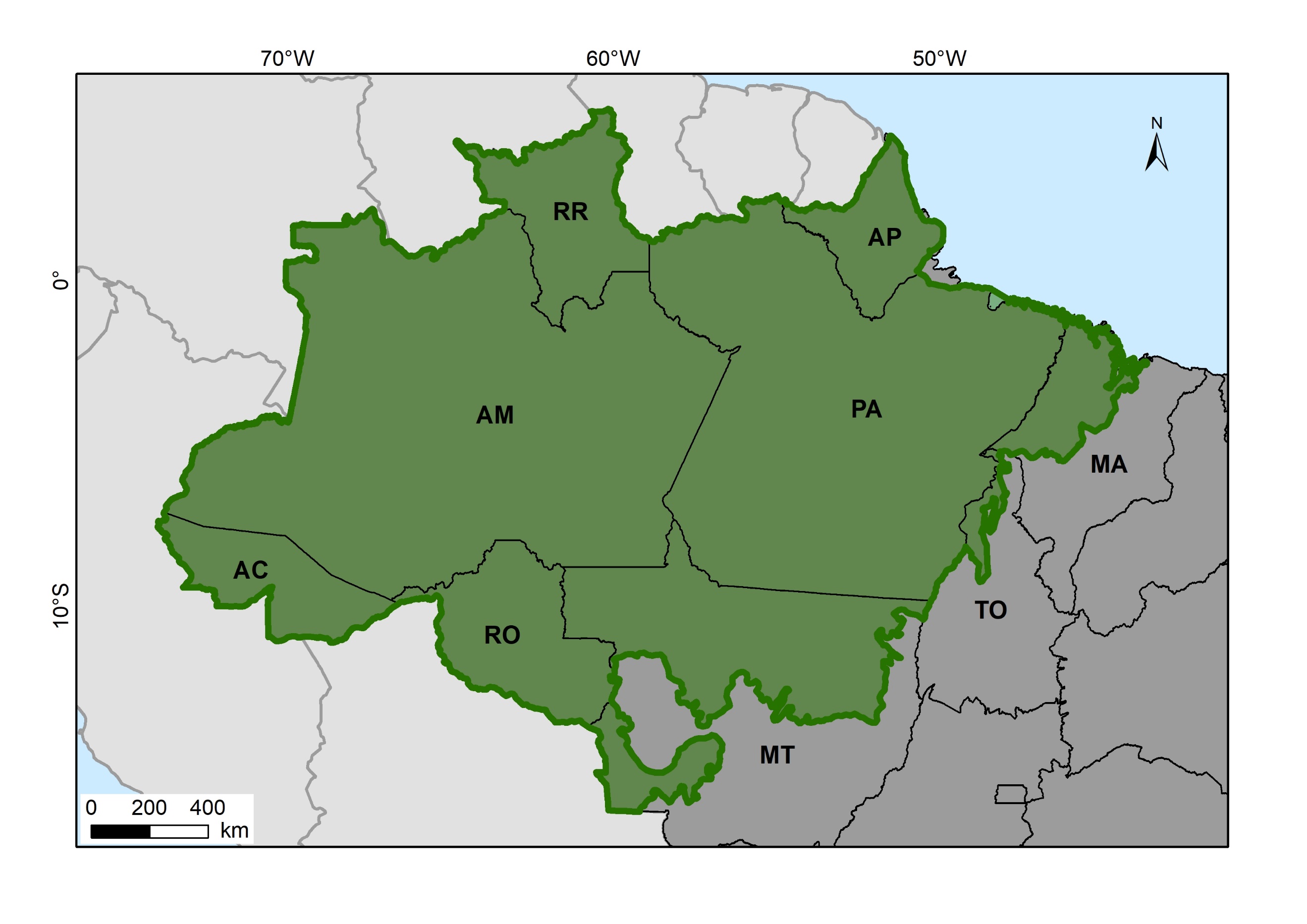
Figure S1. The geographical location of the Brazilian Amazonia biome (the green region with thick green line border). Light grey (thin black) lines delimit Brazilian states (South American countries).

**
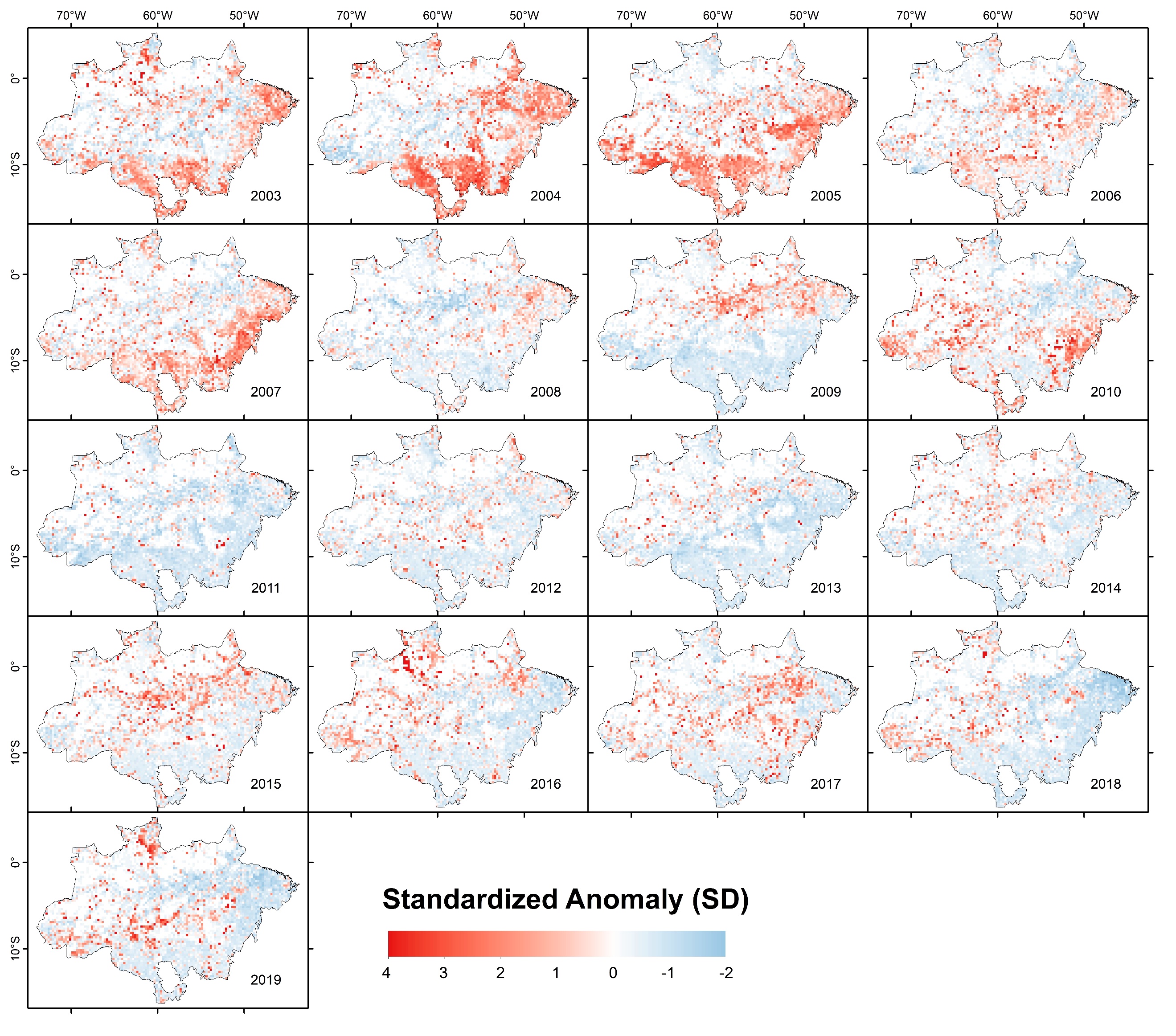
**

**Figure S2.** Fire activity over the BAMZ at 0.25º grid cell, showing annual spatial patterns of standardized anomalies of active fires relative to 2003 – 2019. Positive (≥1 standard deviation) and negative (≤1 standard deviation) standardized anomalies are depicted in red and blue, respectively. We quantified the standardized anomalies for the total annual amount of active fires at each grid cell by their departures from the long-term mean, normalized by the respective long-term standard deviation for 2003 – 2019.

**
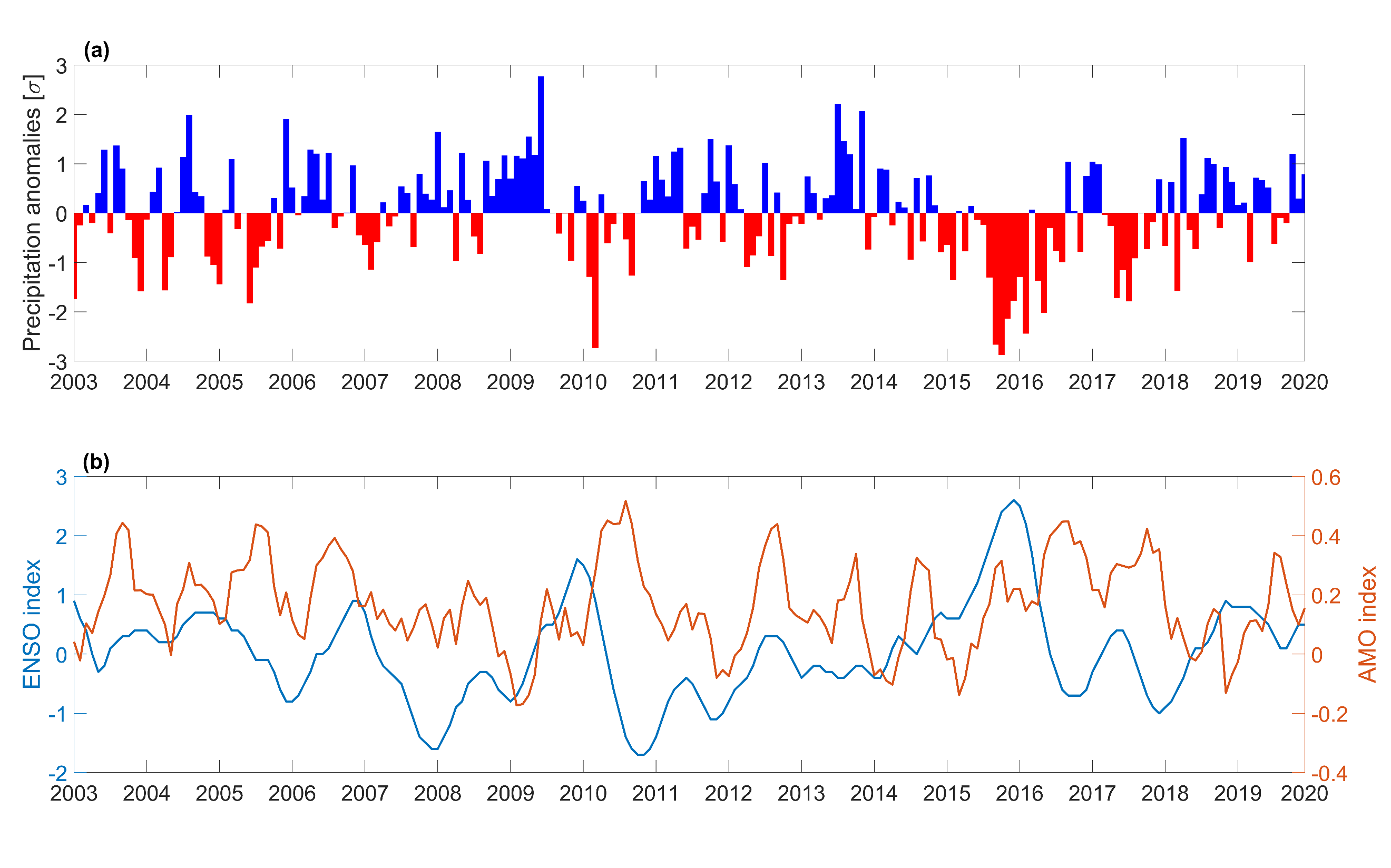
Figure S3.** Droughts patterns over the Brazilian Amazonia, highlighting extreme drought years. Monthly standardized precipitation anomalies during the study period (2003 – 2019) (a); positive anomalies are highlighted in blue and negative ones in red, and the time series of the ENSO (Niño 3.4 SST Index) and AMO (Atlantic Multidecadal Oscillation index) (b), representing the monthly sea surface temperature (SST) anomalies in the Pacific and Atlantic, respectively. Monthly precipitation data were extracted from the Tropical Rainfall Measuring Mission (TRMM) Multisatellite Precipitation Analysis (TMPA) 3B43 Product V7, at 0.25° resolution, https://disc.gsfc.nasa.gov/datacollection/TRMM_3B43_7.html.The Niño 3.4 SST Index and AMO index, which are based on the time series of SST anomalies for the Pacific and Atlantic Oceans, respectively, were obtained from the National Oceanic and Atmospheric Administration (NOAA) at https://www.esrl.noaa.gov. The Niño 3.4 SST Index is calculated from the SST anomalies that are derived from Version 1.1 of the Hadley Center Sea Ice and Sea Surface Temperature (HadISST1.1) in the Niño 3.4 region (5°N-5°S, 120°-170°W) from 1870 to present. The AMO index is derived from Kaplan SST averaged in the North Atlantic over 0–70°N from 1871 to present.


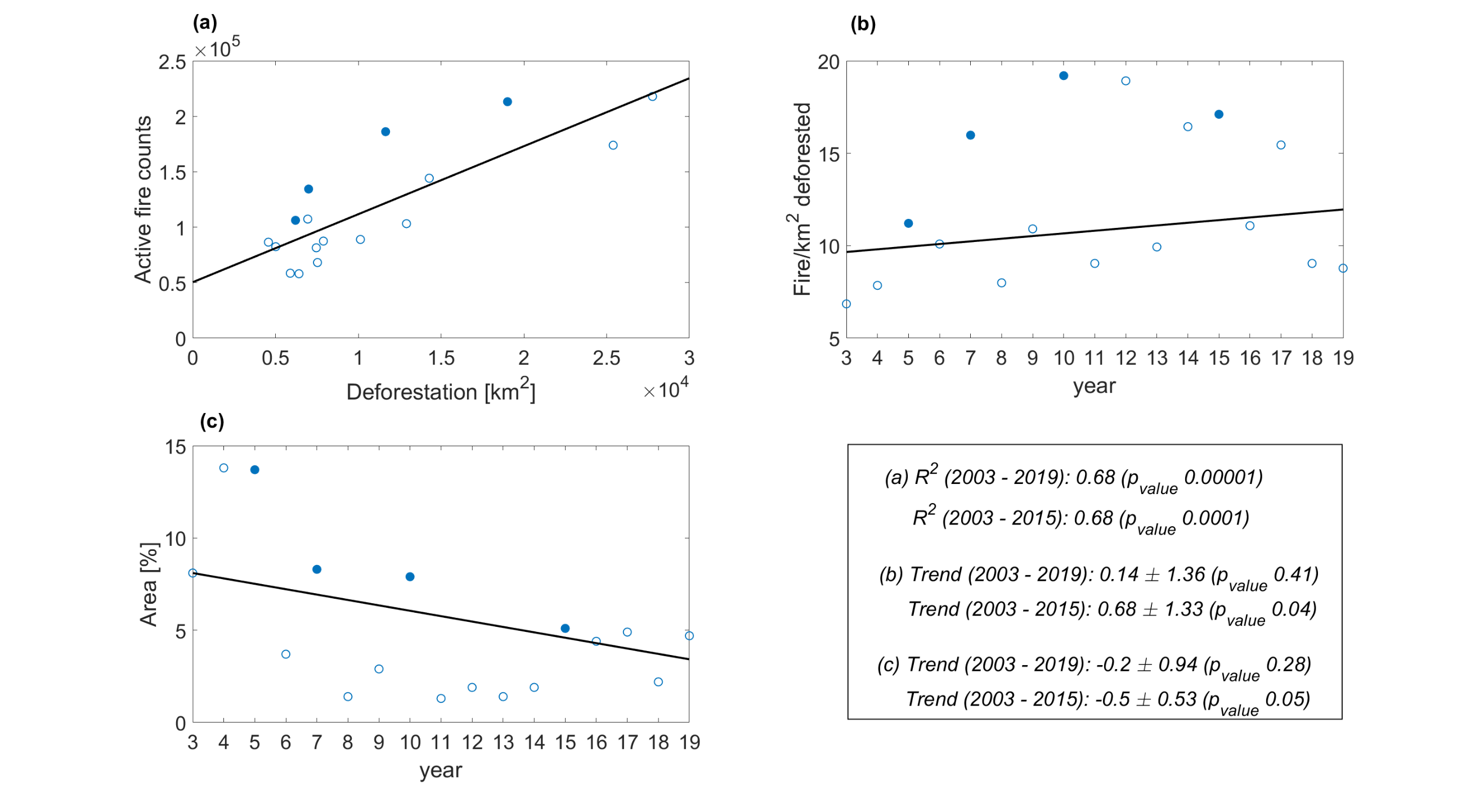
**Figure S4.** (a) Active fire counts over BAMZ as a function of deforestation (km^2^) in 2003 – 2019; (b) Time series of the annual number of active fires per km^2^ deforested from the current database until 2019; (c) Time series of the percent area of the BAMZ with active fire count anomalies in excess of 2 standard deviations using the current database until 2019. Trend significance was analyzed with a two-tailed Mann-Kendall test of significance at the 0.01 level, and trend slopes were estimated using the non-parametric Theil-Sen robust linear regression. Full dots indicate drought years (2005, 2007, 2010 and 2015).


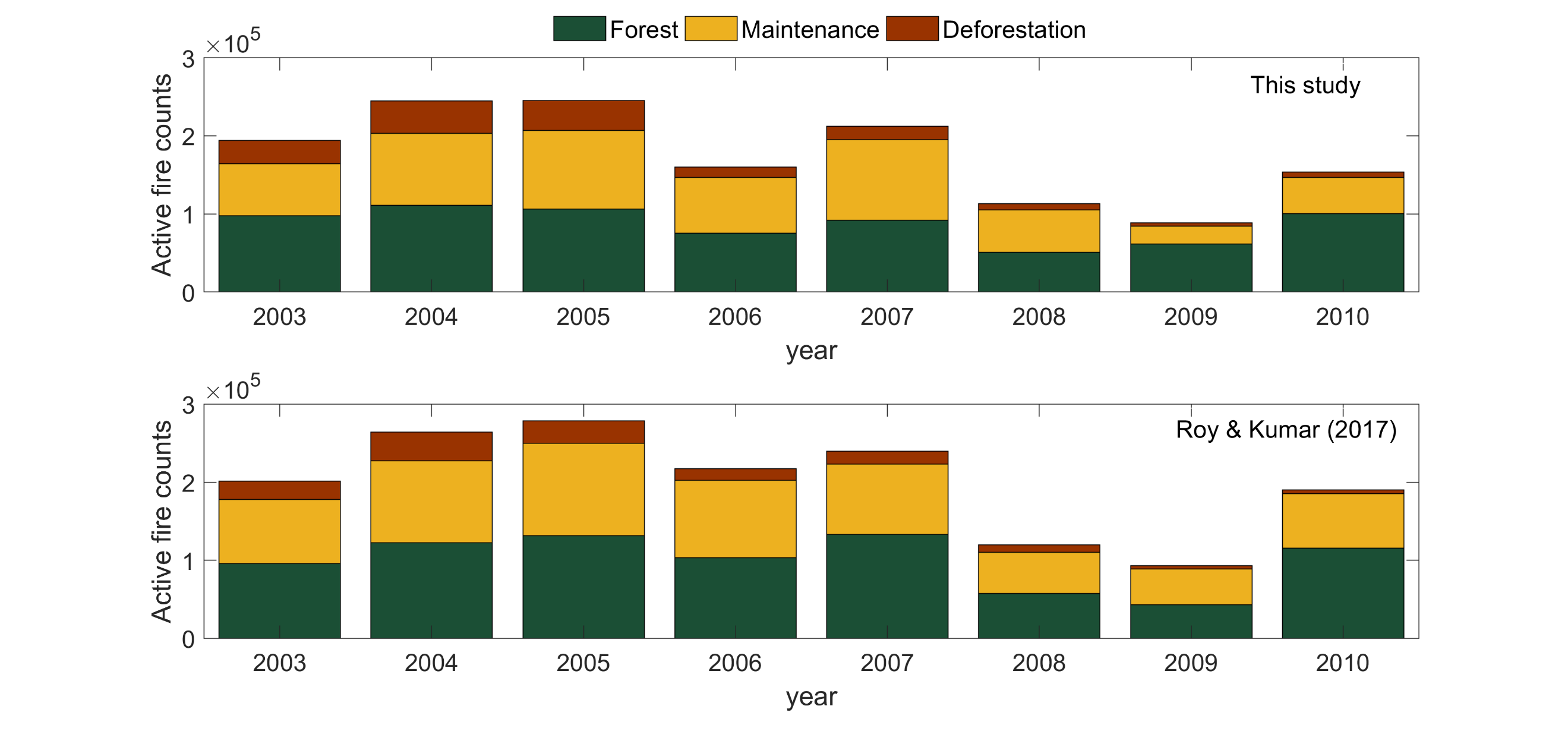


**Figure S5.** Interannual variability of active fires classified as forest fires (green), maintenance fires (orange), and deforestation fires (brown) for the BAMZ during the 2003 – 2010 period: results from this study (AQUA, upper panel) where forest, maintenance and deforestation fires represent 50%, 40% and 10% of total fires are very close to results obtained by Roy and Kumar (2017) (AQUA+TERRA, lower panel) where the contributions are 50%, 41% and 9%.


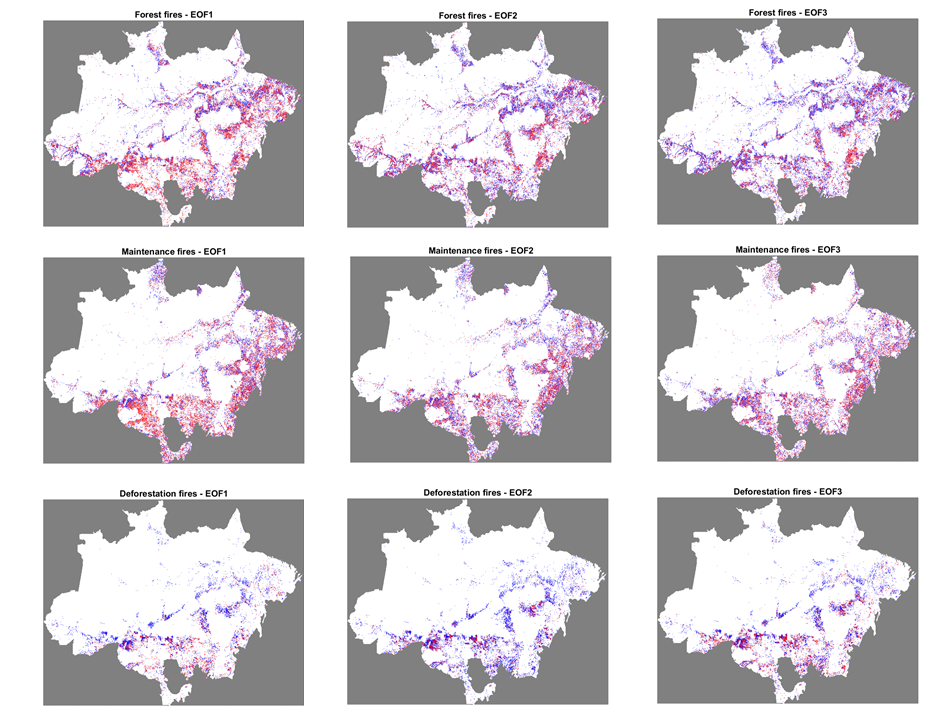


**Figure S6.** Spatial patterns of variability of fire activity in BAMZ as revealed by the sign (positive in red, negative in blue) of the first three leading EOFs (EOF1 – 1^st^ column; EOF2 – 2^nd^ column; EFO3 – 3^rd^ column derived from PCA performed over forest fires (1^st^ line), maintenance fires (2^nd^ line) and deforestation fires (3^rd^ line).
